# Improving the sensitivity of FT-NMR spectroscopy by apodization weighted sampling

**DOI:** 10.1007/s10858-019-00243-7

**Published:** 2019-05-02

**Authors:** Bernd Simon, Herbert Köstler

**Affiliations:** 1 European Molecular Biology Laboratory (EMBL), Structural and Computational Biology Unit, Meyerhofstrasse 1, 69117 Heidelberg, Germany; 2 Department of Diagnostic and Interventional Radiology, University Hospital of Würzburg, Oberdürrbacher Str. 6, 97080 Würzburg, Germany

**Keywords:** Non-uniform sampling, NUS, Fourier transform, Apodization function, Window function, Data acquisition, Nuclear magnetic resonance spectroscopy

## Abstract

Apodization weighted acquisition is a simple approach to enhance the sensitivity of multidimensional NMR spectra by scaling the number of scans during acquisition of the indirect dimension(s). The signal content of the resulting spectra is identical to conventionally sampled data, yet the spectra show improved signal-to-noise ratios. There are no special requirements for data acquisition and processing: the time-domain data can be transformed with the same schemes used for conventionally recorded spectra, including Fourier transformation. The method is of general use in multidimensional liquid and solid state NMR experiments if the number of recorded transients per sampling point is bigger than the minimum required phase cycle of the pulse sequence.

**Electronic supplementary material:**

The online version of this article (10.1007/s10858-019-00243-7) contains supplementary material, which is available to authorized users.

## Introduction

Resolution and sensitivity are the essential quality parameters of high field NMR spectra. Any improvement of one of them without compromising the other is of major importance to allow efficient data analysis. Biomolecules are especially challenging in this respect, since they are prone to strong signal overlap, severe line broadening and low sensitivity. The NMR characterization of biomolecules generally requires the acquisition of a number of multidimensional spectra. The sampling rate of an indirect frequency dimension of these spectra is related to the spectral range to be covered through the Nyquist relation and spectral resolution requirements dictate the total data length of the data recording. In practice, this leads either to long experimental times or compromises in spectral resolution and/or sensitivity. This limitation can be overcome by altering the uniform sampling scheme of the indirect dimension(s), either by coupling the increments of two or more indirect dimensions (Brutscher et al. 1994; Kim and Szyperski 2003; Szyperski et al. 1993) or by recording only a random fraction of the sampling points (Barna et al. 1987; Schmieder et al. 1993, 1994). The first method leads to an effective reduction of the dimensionality by projecting the coupled time domains onto one plane (Freeman and Kupce 2012; Hiller and Wider 2012). For the second method the conventional digital Fourier transformation (DFT) leads to very low quality spectra and thus alternative non-linear processing methods are required to reconstruct the spectrum. For these so-called nonuniform sampling (NUS) methods a number of different sampling schedules (Barna et al. 1987; Hyberts et al. 2010; Mobli et al. 2006; Rovnyak et al. 2004; Schuyler et al. 2011; Holland et al. 2011; Kazimierczuk and Orekhov 2011; Craft et al. 2018) and processing schemes (Chylla and Markley 1993, 1995; Gutmanas et al. 2002; Hoch 1989; Hyberts et al. 2007; Kazimierczuk et al. 2006; Mobli et al. 2012; Orekhov and Jaravine 2011) have been proposed. In biomolecular experiments low sensitivity is a major obstacle. Many of the standard 3D experiments are recorded with a larger number of transients per time domain point than required for signal selection. NUS applications in this so-called sensitivity limited regime are usually implemented by choosing the sampling probability of a point on a sparse uniform grid based on the signal envelope function (Hyberts et al. 2017; Palmer et al. 2015; Rovnyak et al. 2004a, b). Optimal sampling schedules for such experiments and their comparison in terms of sensitivity are discussed controversially (Zambrello et al. 2017, 2018). Common to all of these publications is the implementation of the sampling schedule as a sequence of sampled data points with a uniform number of transient recorded per point interleaved with gaps.

The alternative of acquiring all points on the grid with different number of transients per point has been discussed in the literature (Kumar et al. 1991; Waudby and Christodoulou 2012), but we could not find applications of this method for recording 3D spectra in standard protein or RNA NMR studies. One reason for this might be that fact that in both above references the number of measured transients per time increment is quite large in the example spectra (40 (Kumar et al. 1991) and 256 (Waudby and Christodoulou 2012), respectively) which might suggest that the method is limited to selected 2D applications, but practically prohibitive for 3D or 4D spectra. A second reason could be a missing theoretical derivation of the achievable sensitivity gain, which was previously addressed qualitatively (Kumar et al. 1991) or for a smooth approximations of selected apodization functions (Waudby and Christodoulou 2012). In this contribution we derive a general expression for the sensitivity of a weighted acquisition experiment. If we choose the acquisition weights to follow the apodization function used to process the equivalent conventional data, the achievable sensitivity gain by weighted sampling is determined by the equivalent noise bandwidth of the apodization function, a property that is tabulated in books or reviews of window functions (e.g. (Harris 1978)). Furthermore we examine the dependence of the sensitivity gain on the chosen quantization, e.g. the number of discrete steps used experimentally to approximate the window function. Weighted sampling is very easy to implement experimentally and improves the sensitivity without compromising the quality of the resulting spectra, even for cases of poor quantization, if for example the first half of the points in the indirect dimension(s) are recorded with double the number of transients compared to the second half. This enables considerable improvements of standard 3D experiments as we exemplify for a HNCA for which the weighted scheme results in 50% sensitivity enhancement compared to the conventional dataset recorded with eight transients per point.

## Theory

We assume that we sample the NMR signal on a uniform grid, resulting in time domain data defined by:

1 $$s\left( t \right)={s^e}\left( {k\Delta t} \right)\exp \left( {i\omega k\Delta t} \right)+q(k\Delta t)~$$

where *s*^*e*^*(t)* is the time domain envelope function that modulates the harmonic signal with the offset *ω* and *q(t)* is a white-noise sequence with variance *σ*_*T*_^*2*^. In each indirect time domain of our NMR experiment, we can arbitrarily choose the number of transients *n(t)* = *n*_*0*_*w(t)* recorded for each time point, resulting in a time domain function:

2 $${s_n}\left( t \right)={n_0}w(k){s^e}\left( {k\Delta t} \right)\exp \left( {i\omega k\Delta t} \right)+\sqrt {{n_0}w\left( k \right)} q(k\Delta t)~$$

In the following we assume that all weights *w(k)* > *0* and refer to this as weighted sampling (WS) if the number of transients differ and uniform sampling (US) if *w(k)* = *1* for different time domain data points. We can multiply each time data point with the inverse of the weight *w(t)* resulting in a scaled signal:

3 $${s_{ns}}\left( t \right)={n_0}{s^e}\left( {k\Delta t} \right)\exp \left( {i\omega k\Delta t} \right)+~\frac{1}{{\sqrt {w\left( k \right)} }}\sqrt {{n_0}} q(k\Delta t)$$

where the signal component of the recorded data is independent of the sampling schedule. The noise component scales with the square root of the recorded transients at each data point and is time independent for US. For WS it additionally scales with the inverse of the square root of the weights and is thus time dependent.

For data processing we generally multiply the time domain signal with an appropriate apodization function *h(k)* to avoid truncation artifacts that result from the finite length *t*^*max*^ of the acquisition delay of *M* indirect data points. The peak height in the resulting spectrum obtained by DFT is (Ernst et al. 1987):

4 $$S={n_0}~\mathop \sum \limits_{{k=0}}^{{M - 1}} {s^e}\left( {k\Delta t} \right)h\left( k \right)$$

The noise component of the windowed and scaled transform is given by:

5 $$Q=\sqrt {{n_0}} \mathop \sum \limits_{{k=0}}^{{M - 1}} q\left( {k\Delta t} \right)\frac{{h\left( k \right)}}{{\sqrt {w\left( k \right)} }}\exp \left( { - j\omega k\Delta t} \right)$$

and the expectation value *E* of the noise power is:

6 $$\begin{aligned} E \left\langle\left| Q \right|^{2} \right\rangle = \mathop \sum \limits_{{k = 0}}^{{M - 1}} \mathop \sum \limits_{{l = 0}}^{{M - 1}} E \langle {q\left( {k\Delta t} \right)q^{*} \left( {l\Delta t} \right) \rangle n_{0} } \frac{{h\left( k \right)}}{{\sqrt {w\left( k \right)} }}\frac{{h\left( l \right)}}{{\sqrt {w\left( l \right)} }}\exp \left( { - j\omega k\Delta t} \right)\exp \left( { + j\omega l\Delta t} \right) \\ ~~~~~~~~~ ~ = ~\sigma _{F}^{2} n_{0} \mathop \sum \limits_{{k = 0}}^{{M - 1}} \frac{{h(k)^{2} }}{{w\left( k \right)}}~~ \\ \end{aligned}$$

We note that the effect of WS on the noise power of the spectrum is equivalent to an apodization with the square root of the inverse of the sampling weights. Since DFT and apodization are linear the SNR per unit time is a good indicator to compare the sensitivity of US and WS (Hoch and Stern 1996) as long as *h(k)* and *w(k)* are chosen within the common limits, e.g. are smooth functions with values between zero and one. We define the SNR of the NMR experiment as the ratio of the peak height S to two times the rms of the expectation value of the spectral noise power and the sensitivity (*sens*) as the SNR per unit time (Ernst et al. 1987):

7 $$~SNR = \frac{S}{{2\sqrt {E\left\langle {\left| Q \right|^{2} } \right\rangle } }} = \frac{{\sqrt {n_{0} } }}{{2\sigma _{F} }}\frac{{\sum {s^{e} \left( {k\Delta t} \right)h\left( k \right)} }}{{\sqrt {\sum {\frac{{h\left( k \right)^{2} }}{{w\left( k \right)}}} } }}$$

and

8 $$sens=\frac{{SNR}}{{\sqrt {{T_{tot}}} }}=\frac{{\mathop \sum \nolimits^{} {s^e}\left( {k\Delta t} \right)h\left( k \right)}}{{2{\sigma _F}\sqrt {\mathop \sum \nolimits^{} \frac{{h{{(k)}^2}}}{{w\left( k \right)}}} \sqrt {\mathop \sum \nolimits^{} {t_0}(k)w\left( k \right)} }}$$

where we introduced the total experimental time *T*_*tot*_ as the sum of all acquired transients times the duration of a single experiment *t*_*0*_*(k)*.

From Eqs. (7) and (8) we can calculate the sensitivity gain of WS (subscript w) compared to US (subscript u) by setting *w(t)* = *1* for US:

9 $$\frac{{SN{R_w}}}{{SN{R_u}}}=\sqrt {\frac{{\mathop \sum \nolimits^{} h{{(k)}^2}}}{{\mathop \sum \nolimits^{} \frac{{h{{(k)}^2}}}{{w\left( k \right)}}}}}$$

and

10 $$\frac{{sen{s_w}}}{{sen{s_u}}}=\sqrt {\frac{{\mathop \sum \nolimits^{} h{{(k)}^2}\mathop \sum \nolimits^{} {t_0}(k)}}{{\mathop \sum \nolimits^{} \frac{{h{{(k)}^2}}}{{w\left( k \right)}}\mathop \sum \nolimits^{} {t_0}(k)w\left( k \right)}}} \cong ~~~\sqrt {\frac{{M\mathop \sum \nolimits^{} h{{(k)}^2}}}{{\mathop \sum \nolimits^{} \frac{{h{{(k)}^2}}}{{w\left( k \right)}}\mathop \sum \nolimits^{} w\left( k \right)}}}$$

The approximation (second equal sign) in Eq. (11) is valid if the repetition time of the experiment is long compared to the maximal evolution time and we can replace *t*_*0*_*(k)* by its average value *t*_*0*_.

To investigate the benefit of WS experimentally we chose an approach that builds on the acquisition schemes proposed by Kumar et al. (Kumar et al. 1991) and Waudby and Christodoulou (2012). We choose the weights to correspond to the apodization function used to process the US data.

11 $$n(k)={n_{min}}ceil\left\{ {\frac{{{n_0}}}{{{n_{min}}}}\left| {h\left( k \right)} \right|} \right\}$$

*n*_*min*_ corresponds to the minimum number of steps of the phase cycle in the pulse sequence that is required for proper signal selection. (Note that alternatively we could use the function round instead of ceil and set *n(k)* = *n*_*min*_ if the result of round is equal to zero). We can combine the scaling and apodization multiplications in the processing scheme of the WS data to a single modified window function *hʹ(k)*.

12 $$h^{\prime}\left( k \right)=\frac{{h\left( k \right)}}{{w\left( k \right)}}$$

**Fig. 1:**
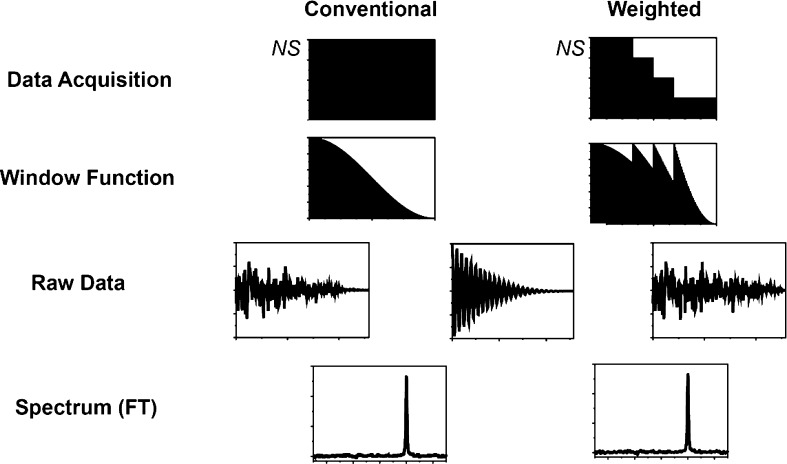
Schematic representation of the weighted sampling scheme. In the conventional data acquisition the number of transient is uniform for all indirect data points and the window function is a smooth curve. In apodization weighted sampling, the window function is moved to the data acquisition as a step function reducing the number of scans with acquisition time. The data are multiplied with a window functions *hʹ* that compensate the discrete jumps in the acquisition setup. As a result, the signal content in the raw data is indentical for both methods after apodization and before FT (center). The noise content is different: in uniform sampling, the noise amplitude is uniform before the window multiplication and thus scales linearly with the window function. In weighted sampling, the noise amplitude is proportional to the square root of the number of scans recorded at each time point which corresponds to the multiplication with the square root of the window function. As a result, the signal part of the resulting spectrum is identical, while the noise content in the weighted spectrum is higher. The time savings for the weighted experiment, which corresponds to the ratio of the total number of scans of the two acquisition schemes, leads to a sensitivity improvement for the weighted scheme

The modified acquisition and processing scheme is depicted schematically in Fig. 1.

If there is more than one indirect dimension, we obtain the sampling scheme by multiplying the window functions of each dimension, so for a 3D experiment:

13 $$n(k,l)={n^{min}}ceil\left\{ {\frac{{{n_0}}}{{{n^{min}}}}{h_k}\left( k \right){h_l}\left( l \right)} \right\}$$

respectively. The function(s) *w(k,l)* and *hʹ(k,l)* are defined accordingly and a generalization to higher dimensions is straightforward.

For large ${n_0}$, the discrete steps in *n(k)* approach a continuous function and *w(k)* = *h(k)*. In this limit we get:

14 $$\mathop {{\text{lim}}}\limits_{{{n_0} \to \infty }} \frac{{SN{R_w}}}{{SN{R_u}}}=\sqrt {\frac{{\mathop \sum \nolimits^{} h{{(k)}^2}}}{{\mathop \sum \nolimits^{} \left| {h\left( k \right)} \right|}}}$$

and

15 $$\mathop {{\text{lim}}}\limits_{{{n_0} \to \infty }} \frac{{sen{s_w}}}{{sen{s_u}}}=\frac{{\sqrt {M\mathop \sum \nolimits^{} {h^2}\left( k \right)} }}{{\mathop \sum \nolimits^{} \left| {h\left( k \right)} \right|}}$$

The window function *h(k)* is generally decaying from *h(0)* = *1* to *h(t*_*max*_*)* ~ *0* and thus the SNR ratio (Eq. 14) is generally smaller than one. However, the sensitivity of WS is larger than the sensitivity of US recorded with the same *n*_*0*_ because the time reduction of the experiment over compensates this loss in SNR. If both experiments are recorded for the same time, the weighed experiment will always exhibit higher SNR.

An eminent feature of WS is the easiness of SNR comparison to a corresponding US dataset. If we chose the weighting to correspond to the apodization function, the sampling scheme is tailored to the processing scheme of the US data. Apodization weighted sampling leads to experimental data, where the signal component of the FID is scaled with the window function, while the noise component of the FID is scaled with the square root of the window function. This leads to a smaller noise dampening of high frequency components in the weighted spectrum compared to the conventional spectrum and thus to a higher noise rmsd (see Fig. S1). SNR and sensitivity (Eqs. 9 and 10) are good indicators for the ability to distinguish signals from noise in the resulting spectra (Zambrello et al. 2018; Hoch and Stern 1996), the more so as we compare spectra with identical line-shapes.

Please note that we have not made any assumptions about the signal envelope in the derivation of the results. SNR and sensitivity of the WS experiment compared to its US counterpart depend on the apodization function only. Properties of different window functions used for signal processing have been examined in a number of publications (e.g. (Harris 1978)). Typical figures of merit calculated for the different window function correspond directly to the most important parameters for the weighted sampling scheme: the time saving for WS corresponds to the mean of the elements of the window function and is called the coherent gain. The sensitivity advantage of WS over US in the limit of large *n*_*0*_ (Eq. 15) corresponds to the square root of the equivalent noise bandwidth of the window function. Other important parameters for the NMR signal as the suppression of truncation artefacts (highest side-lobe level) and the line broadening (which is closely related to the 3.0-dB or 6.0-dB band-width) are not affected by the weighted sampling compared to uniform sampling and will not be discussed here further.

## Experimental details

Experiments have been conducted on Bruker Avance III and DRX consoles using different software version including TopSpin (Bruker) 1.3, 2.1 and 3.5. For the implementation the acquisition loop in the pulse program is modified. Instead of looping with the constant acquisition parameter *NS* (number of scans) for each indirect acquisition point, we loop using a variable counter that is defined in a counter list (Supplement). We use a python script to generate the variable counter list (Eq. 11 or 13), which also outputs a list of multipliers used for processing the acquired data, e.g. the functions *w(k)*^−*1*^ and *hʹ(k)* (Eq. 12). We choose to write one entry for each recorded FID in these lists. For processing, we use a C-program that multiplies each time domain FID with the corresponding number *w(k)*^−*1*^ or *hʹ(k)* and use the modified raw data for further processing either within TopSpin or NMRPipe (Delaglio et al. 1995).

For apodization we used the first lobe of the cosine function:

16 $$h\left( k \right)=co{s^\alpha }\left( {\frac{\pi }{2}\frac{k}{{M - 1}}} \right)$$

the exponential function:

17 $$h\left( k \right)={e^{ - \alpha \frac{k}{{M - 1}}}}$$

and the Gaussian function:

18 $$h\left( k \right)={e^{ - \frac{1}{2}{{\left( {\alpha \frac{k}{{M - 1}}} \right)}^2}}}$$

The cosine (Eq. 16) is implemented in NMRPipe as function SP with the parameters off = 0.5, end = 1.0 and pow = α. In TopSpin it corrspeonds to the functions sin (α = 1) and qsin (α = 2) with the parameter ssb = 2. The exponential corresponds to the NMRPipe and TopSpin functions EM with the parameter lb = αΔf/π where Δf is the spectral resolution. The Gaussian is equivalent to the NMRPipe function GM with the parameters g1 = g3 = 0 and g2 = 0.375αΔ f.

SNRs were calculated as ratio of the average peak intensities of a number of isolated signals in the corresponding spectra and the noise root mean square deviations (rmsds) using NMRView (Johnson et al. 1994). The noise rmsd is estimated as the standard deviation of the data points in an area of the spectrum without signals. Alternative routines for calculating the spectral noise in TopSpin (routine sino for 1D traces of the spectrum) or NMRPipe (routine showApod) were also tested and give qualitatively similar results, though the exact SNR differ slightly due to the differences in the selected data points and the equations used to calculated the noise rmsd. Exact experimental times were extracted from the audit file for each dataset and used to calculate the sensitivities.

## Results and discussion

### 2D 1H-15N HSQC spectra recorded with conventional and apodization weighted sampling

An illustrative example for the improvement in sensitivity is obtained, if we record 2D spectra with a window function corresponding to the first lobe of a squared cosine function (Eq. 16 with α = 2).

**Fig. 2:**
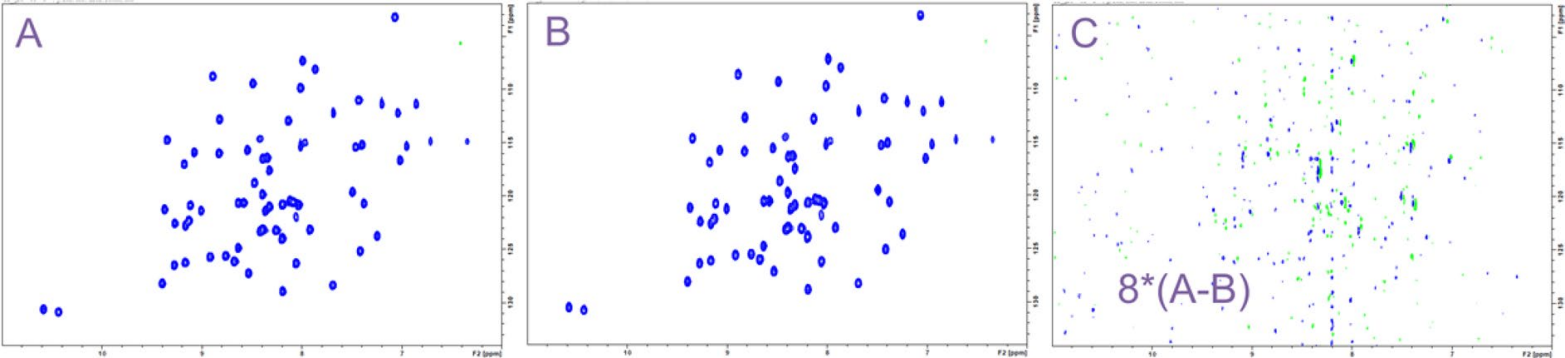
Comparison of ^15^N, ^1^H sensitivity enhanced HSQC spectra of GB1 recorded on a Bruker DRX500 with TopSpin1.3. The experiments were acquired with maximum acquisition times of 38.4 and 136 ms, spectral widths of 1667 and 7508 Hz using 64 and 1024 complex points for ^15^N and ^1^H respectively. **a** Uniform sampling with *NS* = 16 acquired in 36 min. The data are processed with the apodization function qsin (Eq. 18). The average peak intensity is 32.1 ± 2.8 and the noise rmsd is 0.0179. **b** Weighted sampling with *NS* reduced from 16 to 1 in integer steps according to Eq. (3) acquired in 19 min. Data processing is identical to the uniformly sampled spectrum, except that no window function is used in the indirect dimension. The average peak intensity is 32.2 ± 2.8 and the noise rmsd is 0.0202. **c** Difference of the uniform and weighted spectra (A-B). The contour levels are reduced by a factor of 8 compared to the spectra in **a** and **b**. The experimental SNR and sensitivity ratios are 0.889 and 1.219 (weighted **b** vs. uniform **a**)

The numerator and denominator in Eqs. (15) are:

$$\mathop \sum \nolimits^{} h=\frac{2M}{\pi }\mathop \smallint \limits_{0}^{{{\raise0.7ex\hbox{$\pi $} \!\mathord{\left/ {\vphantom {\pi 2}}\right.\kern-0pt}\!\lower0.7ex\hbox{$2$}}}} co{s^2}\left( t \right)dt~=\frac{1}{2}M~~~~and~~~\sqrt {M\mathop \sum \nolimits^{} {h^2}} ={\left( {\frac{2M^2}{\pi }\mathop \smallint \limits_{0}^{{{\raise0.7ex\hbox{$\pi $} \!\mathord{\left/ {\vphantom {\pi 2}}\right.\kern-0pt}\!\lower0.7ex\hbox{$2$}}}} co{s^4}\left( t \right)dt} \right)^{1/2}}=\sqrt {\frac{3}{8}~}M$$

where we have replaced the sum over all time points by an integral from 0 to t^max^. Since the average of the apodization function is ½, we can record the weighted sampling experiment with twice the number of scans for the first FID compared to the uniform scheme in the same total experimental time. This results in a doubling of the signal amplitude *S* (Eq. 4). If we are in the limit of large *n*_*0*_ and don’t use a correction function *hʹ* the SNR ratio is (3/4)^1/2^ = 0.866 (Eq. 14) and the sensitivity gain of the weighted scheme is 2(3/8)^1/2^ = 1.225 (Eq. 15 and (Waudby and Christodoulou 2012)). Experimentally, we compare 2D sensitivity enhanced ^15^N, ^1^H -HSQC (Fig. S2) spectra of the B1 immunoglobulin-binding domain of the streptococcal protein G (GB1) (Gronenborn et al. 1991) with standard and weighted sampling. The experimental sensitivity gain matches the theoretical prediction and the quality of the two spectra is virtually identical (Fig. 2). Note that the SNR and sensitivity ratios calculated for the digitized window function (Eqs. 9 and 10) used in Fig. 2b are 0.890 and 1.219.

### Comparison of spectra with and without processing correction factors

The example in the previous paragraph and similarly previously published results (Waudby and Christodoulou 2012) illustrate the feasibility of the sampling strategy and show the excellent agreement of the theoretically expected and observed sensitivity improvements. In our setup with a *t*_*max*_ of 38.4 ms, the signal decay during the evolution of the indirect dimension is ~ 20% for the GB1 sample with amide nitrogen T2 times of ~ 195 ms at 500 MHz and 25 °C. The GB1 spectra shown are processed without using correction *hʹ(k)* or scaling factors *w(k)*^−*1*^ which does not introduce any noticeable spectral artefacts due to the smooth decay of *n(k)* and the rather uniform peak shape for all for GB1 HSQC signals. To examine potential artefacts introduced by the sampling method, we chose a different protein sample—the 134 residue BRDT bromodomain 1 (Miller et al. 2016) (BD1) at 0.2 mM concentration. For BD1 we observe an average T2 of ~ 64 ms for the structured backbone amide nitrogen atoms and T2s of ~ 500 ms for residues in the flexible N- and C-termini at 25 °C and 600 MHz and thus much more variable peak shapes.

**Fig. 3:**
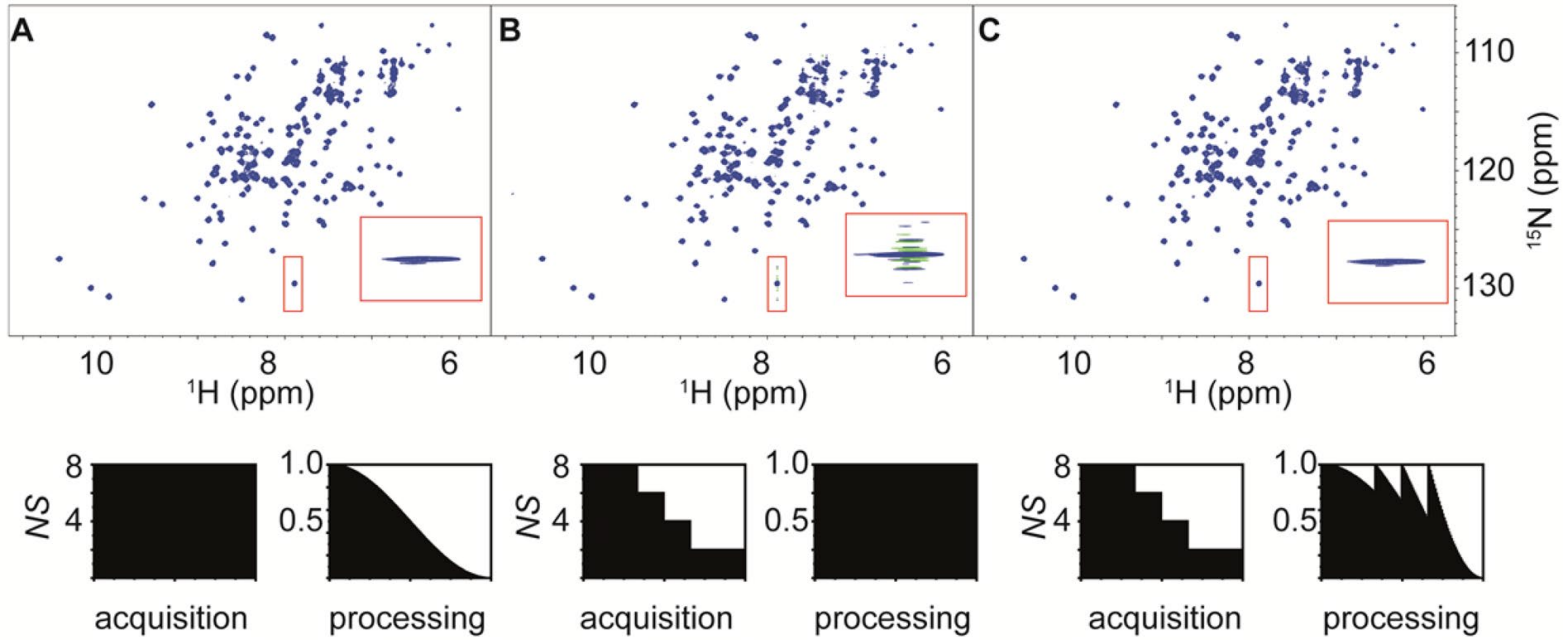
Comparison of ^15^N, ^1^H water-gate HSQC spectra of BD1 recorded on a Bruker AVIII 700 with TopSpin3.5. Data were acquired with acquisition times of 64/90 ms, spectral widths of 1992/11,261 Hz and 128/1024 complex points for the ^15^N/^1^H dimensions. **a** Uniform data acquisition with NS = 8 and processed with a squared cosine window function recorded in 39 min. **b** Weighted data acquisition recorded in 24 min and processed with no window function in the ^15^N dimension. The processing of the weighted dataset without apodization leads to significant side-lobes most prominently visible for the flexible C-terminal residue highlighted in the inset. **c** Same data as in **b**, employing the correction *hʹ* (Eq. 12) for data processing. The spectrum is virtually identical to the spectrum obtained from the uniform dataset

First we ran a standard ^15^N, ^1^H HSQC pulse sequence (Fig. S3) from the Bruker pulse program library (fhsqcf3gpph) with *NS* = *8* for the reference spectrum and reduced the number of scans in steps of *ns*_*min*_ = 2 in the weighted acquisition (Eqs. 11 and 16). The four step reduction of the number of scans in the indirect dimensions leads to visible artefacts if the data are processed without window function in the indirect dimension (Fig. 3b). These digitization artefacts are fully removed if the data are processed with the correction functions (Eq. 12), leading to a spectrum of similar quality as for uniformly acquired data (Fig. 3a, c).

**Fig. 4:**
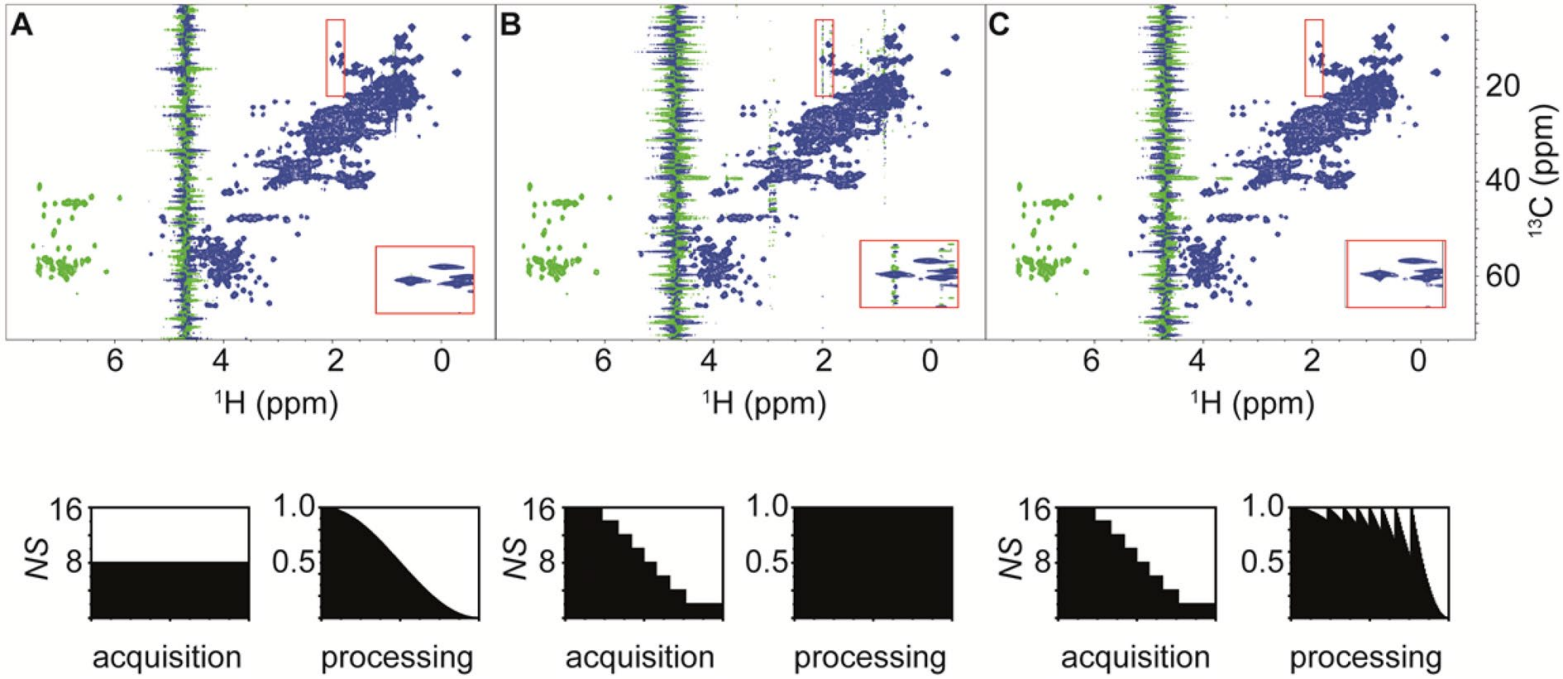
Comparison of ^13^C, ^1^H HMQC spectra of BD1 in 95%H_2_O/5%D_2_O. Data were acquired with acquisition times of 10.4/104 ms, spectral widths of 12,500/9804 Hz and 128/1024 complex points for the ^13^C/^1^H dimensions at a ^1^H frequency of 700 MHz. **a** Uniform data acquisition with *NS* = *8* in 47 min and processed with a squared cosine window function. **b** Weighted data acquisition data acquisition with n_0_ = 16 scans for the first FID reduced in eight steps to two scan recorded in 53 min and processed with no window function in the ^13^C dimension. **c** Same data as in **b**, employing the correction *hʹ* (Eq. 12) for data processing. The resulting spectrum is virtually identical to the uniform data

To see if the results also transfer to experiments with higher stability requirements, we recorded ^13^C, ^1^H-HMQC (Fig. S4) spectra on the same sample (e.g. in 95% H_2_O, 5% D_2_O buffer solution). In this experiment, the water suppression is achieved by low power irradiation on the water resonance frequency during the recycling delay and two step phase cycles of the 90° pulses surrounding the ^13^C evolution period. Also in this case, the spectral quality of the conventional experiment and the experiment with the apodization weighted sampling scheme are identical (Fig. 4a, c), if the correction is used in the processing scripts, while omitting the correction function in the indirect dimensions leads to pronounced wiggles in the spectrum (Fig. 4b).

### Calculated and experimental sensitivity gain

**Fig. 5:**
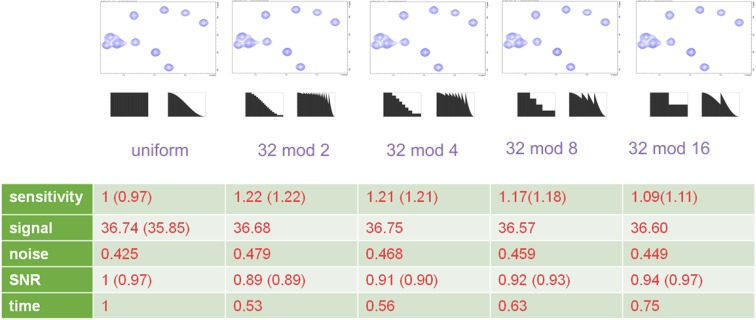
Comparison of the calculated and experimental sensitivity gain of the weighted acquisition ^15^N, ^1^H water-gate HSQC as a function of the number of steps. The spectral parameters are identical to Fig. 3. The calculated signal intensity for the different spectra corresponds to the average peak height of 79 well resolved resonances. The spectral noise was estimated as the standard deviation of the intensities in a rectangular area (^1^H 10.0–12.5 ppm ^15^N 107–119 ppm) outside the signal range. The values in parentheses correspond to the values of a repeated data acquisition for the uniform sampling scheme and to the theoretical values (Eqs. 9 and 10) for the weighted sampling. The data quality is identical for all spectra

In the previous sections, we have shown that the spectra of the apodization weighted sampling scheme are of similar quality to spectra with conventional acquisition and usage of the corresponding window function in the processing script. Next we want to examine the experimental sensitivity gain for different setups and compare the results with the theoretical prediction. First we compare the sensitivity gain as a function of the number of steps of *n(k)*. For this purpose we recorded the ^15^N, ^1^H HSQC water-gate experiment with *n*_*0*_ = 32 scans and reduced the number of scans in steps of 2, 4, 8 or 16, respectively. For comparison we ran a conventional experiment with *n*_*0*_ = 32 before and after the acquisition of the four apodization weighted experiments (Fig. 5). All spectra appear virtually identical after processing showing average signal intensities of 36.5 ± 0.3 (a.u.). The noise amplitude increases from 0.425/0.428 in the conventional experiments to 0.479 in the experiment where *n(k)* is reduced in 16 steps from 32 to two scans. This corresponds to a SNR reduction of 10% with a time reduction of 47% when these experiments are compared. For the rather smooth 16 step reduction, the sensitivity gain is 24% when compared to the average sensitivity of the conventional experiments and corresponds to the theoretical value for the smooth window function within the experimental error. (The difference in sensitivity of the two conventional experiments is 3%). The sensitivity gain gradually reduces with a smaller number of steps, but even in the case of a single step, e.g. recording the first half of the indirect dimension with 32 and the second half with 16 scans, we achieved 11% sensitivity improvement. The experimentally observed SNR and sensitivity ratios correspond to the theoretically calculated values within the experimental error. The results are summarized in Fig. 5. For the comparison we recorded data with the same *n*_*0*_ to have equal signal intensity in all datasets. Similar results for the relative SNR and sensitivity are achieved for *n*_*0*_ reduced to 16, 8 and 4 and a stepsize of 2, respectively.

The sensitivity gain is largely independent of the chosen spectral resolution determined by the maximal evolution time of the indirect dimension *t*^*max*^. This is because the dependence of the sensitivity of US and WS data on *t*^*max*^ are identical and cancels if we calculate the ratio. Comparing HSQC spectra with the number of complex points in the indirect dimension ranging from 24 to 384 (e.g. *t*^*max*^ ~ 0.2T_2_–3T_2_) result in a sensitivity gain of ~ 18% and ~ 22% for NS = 8 and NS = 32 respectively. For experiments with long indirect evolution times, the ratio of the total time of the conventional and weighted experiment is increasingly bigger than the ratio of the total number of scans of the two experiments, since the incremented time delay cannot be neglected and thus the sensitivity gain in these cases is slightly higher (Eq. 10). In the present experiment with a repetition time of one second the effect is small, but it would become more significant for experiments with long *t*^*max*^ and short recycling delays as for example the SOFAST HMQC.

To further test the theoretical predictions, we ran weighted spectra with different cosine, exponential and Gaussian window functions. The parameters of the window functions were chosen to achieve similar suppression of the highest truncation side lobe. Any improvement in side-lobe suppression also leads to an increased line broadening of the signals. The window functions which decay faster and thus achieve stronger side-lobe suppression also reduce the signal intensities and lead to a slight decay of the SNR in the conventionally recorded spectra. For the apodization weighted sampling, this SNR reduction is more pronounced, but the time saving over compensates this effect, leading to an increase in sensitivity with stronger windowing. The sensitivity gain of the apodization weighted experiment for a exponential window function increases from 14 to 43% if the scaling of the last point is increased from *e*^−2^ to *e*^−4^ (corresponding to a line broadening of 10–20 Hz in the present setup.) Similar trends are observed for Gaussian and sine bell windows (Table 1).

**Table 1:** Sensitivity comparison of ^15^N, ^1^H water-gate HSQC spectra processed and recorded with different window functions

Function	Acquisition	Processing	Relative sensitivity weighted/uniform		
			Reference A	Reference B	Calculated
Cosine α = 2			1.19	1.18	1.22
Cosine α = 1			1.13	1.10	1.11
Exponential α = 2			1.18	1.18	1.14
Exponential α = 3			1.31	1.28	1.28
Exponential α = 4			1.49	1.44	1.43
Gaussian α = 2			1.14	1.12	1.11
Gaussian α = 2.5			1.24	1.21	1.20
Gaussian α = 3			1.34	1.30	1.29

Data were acquired with acquisition times of 64 and 90 ms, spectral widths of 1992 and 11,261 Hz and 128 and 1024 complex points for the ^15^N/^1^H dimensions and *n*_*0*_ = 32 scans for the first indirect point. The window functions are defined in the experimental details section. The graphical insets show the number of transients recorded for each data point and the step correction used to process the data. The sensitivities were determined relative to conventional experiments recorded at the beginning and end of the series of experiments and processed with the corresponding window functions (Reference A and B) and are compared to the calculated values (Eq. 16)

### Higher dimensional spectra

**Fig. 6:**
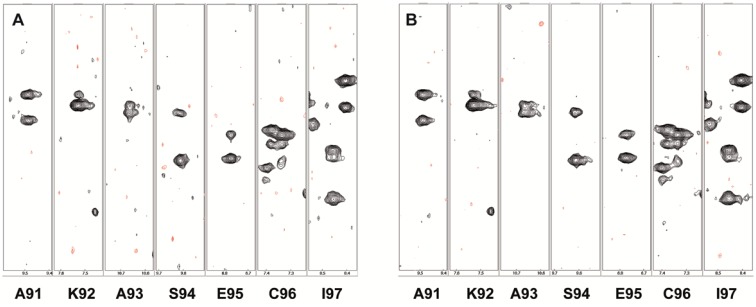
Comparison of 700 MHz 3D-HNCA spectrum of BD1. **a** Uniform sampling with *NS* = *8* and 46 and 60 complex points for ^15^N and ^13^C evolution and spectral widths of 28.1 ppm and 35.5 ppm recorded in 24.7 h. Prior to FT the data have been apodized with a squared cosine function in both indirect dimensions. **b** Apodization weighted acquisition with *n*_*0*_ = 32 and *n*_*min*_ = 2 recorded in 26.6 h and processed with the appropriate correction function. The quality of the two spectra is virtually identical. To compare the SNR and sensitivity we picked 128 isolated peaks and determined average peak intensities of 5.4 ± 3.5 and 22.4 ± 14.7 for the uniform and weighted spectra and a SNR of 10.2 and 16.0 respectively. The recording of the weighted dataset took 7.5% longer compared to the conventional sampling for this setting resulting in a total increase of 52% in sensitivity

Increasing the dimensionality of a spectrum results in a large growth of the number of the indirect sampling points. This limits the practicability of recording uniformly sampled data to three or four dimensions. Nevertheless, many 3D and some 4D datasets of biomolecules are recorded with more than the minimally required number of scans which is equal to two for many standard experiments. In such cases the weighted sampling scheme is applicable to all indirect dimensions. The total time saving and the sensitivity gain for a nD spectrum correspond to the product of the values obtained for each of the n dimension. If we use a squared cosine lobe as window function in each indirect dimension, the experimental time reduces by a factor of 4 for a 3D and 8 for a 4D experiment when compared to a conventionally sampled dataset with the same initial *n*_*0*_ or equivalently *n*_*0*_ can be increased by the factors 4 or 8 for an equivalent experimental time. Since the sensitivity gain is independent of the signal shape, it equally holds for periods of constant or real time chemical shift evolution and is—as in the 2D case—mostly independent of the number of points used in each of the indirect dimensions (within the limits that are used in practice.) As an example we compared a conventional HNCA experiment recorded on the BD1 sample with eight scans to its weighted counterpart starting with 32 scans. The experimental time for the weighted experiment is 7.5% longer in the present setup since we use the ceiling approximation (Eq. 11) to calculate the number of scans for each point. The pulse program is from the Bruker standard library (hncagp3d) and we used the full original 16 step phase cycle resulting in an incomplete phase cycle for most indirect data points. The signal selection in this experiment is achieved by echo-antiecho gradients in a single scan. The chosen minimum two step phase cycle selects magnetization transferred to ^13^Cα. All other steps in the phase cycle remove pulse imperfections and could be replaced by appropriate pulsed field gradients. The resulting spectra are of similar quality, while the sensitivity of the weighted dataset is 50% higher (Fig. 6).

## Discussion

Most experimental NUS approaches focus on skipping data points on a uniform sampling grid, while recording the remaining points with a uniform weight. This is the only feasible approach if the data acquisition is resolution limited, e.g. in cases where the SNR is high and the experiment is recorded with the minimum number of scans required for signal selection. In this case NUS achieves a reduction of measurement time for a targeted spectral resolution. A large number of 3D, most 4D and all experiments with higher dimension fall in this regime. Despite the steady increase in spectrometer performance there are, however, still 2D and 3D applications where the experiment is SNR limited, e.g. more scans per sampling point are required to achieve an acceptable spectral quality. In this case the major benefit of NUS is to improve the sensitivity for experiments with non-constant signal envelopes by adjusting the sampling density to the signal envelope. The characterization and quantification of the achievable sensitivity enhancements in dependence of the time domain signal envelope, the sampling schedule and the processing scheme are addressed in a number of recent publications with partially controversial results. While for example the iSNR metric introduced by Rovniak et al. (Palmer et al. 2015) suggests that no sensitivity improvements is achieved for a constant signal envelope, Zambrello et al. (2018) report an unexpected sensitivity gain in a HNCACB experiment recorded with a semi-constant and constant indirect time domain by NUS validated by the iROC analysis. One of the problems for a stringent characterization of sensitivity and spectral quality stems from the fact that the methods to reconstruct artefact free spectra from the gapped time-domain data are inherently non-linear and the simple and robust definition of sensitivity as the ratio of signal amplitude to noise rmsd per unit time that is widely used to characterize the quality of spectra obtained by DFT leads to wrong results (Hoch and Stern 1996). This difficulty is not present if the data are acquired with a weighted sampling scheme where the desired sampling density is achieved by modulation of the number of transients recorded per time-domain point without gaps. The WS approach should allow disentangling the NUS improvements achieved by the sampling schedule on the one hand and the non-linear processing scheme on the other hand.

One easy implementation of weighted sampling is the shifting of the apodization function used in the processing script of the corresponding conventionally recorded data as weight for the number of scans during data acquisition. The signal content of such data is identical to the windowed conventional dataset and thus the signal parts of the processed spectra are identical. The random noise component is windowed with the square root of the chosen apodization function. This is achieved by modulating the number of recorded transients per time point with an integer multiple of the apodization function at each point. If each time domain data point is scaled by the inverse of its own weight, the signal component of each FID is identical to the corresponding FID in US, while the noise rmsd is increasing for points with smaller weights. After this scaling the data can be processed similar to a conventional dataset, thus by windowing with either the apodization function used to tailor the acquisition or any alternative scheme. It should be pointed out however, that the widely-used method of linear prediction is expected to perform considerably worse or fail completely since the noise content of the last time domain points is high. If the processing scheme of the uniform data includes linear prediction it is advisable to increase the time domain in the corresponding dimension when recording the weighted data and remove the linear prediction from the processing. Except for this adjustment, we omit the discussion of the optimal setup of the experiment in terms of *t*^*max*^, resolution and apodization function and we assume that all these factors have been optimized for the conventional experiment. Independent of the details of the setup and the signal envelope the shifting of the apodization function to the data acquisition always increases the sensitivity of the experiment.

The achievable sensitivity gain and the reduction in experimental time depend on the chosen apodization function only and the corresponding values are tabulated in the literature for many window functions used for signal processing. The sensitivity gain corresponds to the square root of the equivalent noise bandwidth and the time reduction to the coherent gain of the chosen apodization function. These values correspond to the maximum sensitivity gain which is achievable if the window function is sampled smoothly, which is the case for a setup where the first data points are sampled with a large number of scans and the reduction occurs in many small steps. In the common experimental settings with a medium number of scans the direct DFT of the raw data introduce noticeable spectral artefacts which are fully removed if the data are first corrected by the modified window function. For commonly used window functions the sensitivity increases more than 20% per dimension leading to approximately 50% sensitivity increase in standard 3D experiments. A small number of steps in the weighted sampling reduce the advantage of the weighted sampling gradually, but even in a one-step case the sensitivity gain is still more than 10% per dimension. This sensitivity gain comes virtually at no costs, since the data processing is identical to the processing of conventional datasets.

## Electronic supplementary material

Below is the link to the electronic supplementary material.

Supplementary material 1 (DOCX 917 KB)
