# Supplementary material for: Improving the sensitivity of FT-NMR spectroscopy by apodization weighted sampling
Source: J Biomol NMR. 2019 May 2;73(3):155–65. doi: 10.1007/s10858-019-00243-7 (PMC6525709; doi:10.1007/s10858-019-00243-7)
Supplement: Supplementary file 1 — Supplementary material 1 (DOCX 917 KB) [file 10858_2019_243_MOESM1_ESM.docx]

**Supporting Information**

Improving the sensitivity of FT-NMR Spectroscopy by apodization weighted sampling

Bernd Simon* and Herbert Köstler

**Experimental Details**

For the experimental implementation, the data acquisition command of the pulse program has to be modified. Most pulse programs use the combined *go* command which starts acquisition, increments the pulse and acquisition phases and loops *ns* times to the defined starting point. This combined command is replaced by separate commands starting acquisition (*goscnp*), incrementing all pulse and acquisition phases appearing in the phase list (*3m ipp1 ...ipp31*) and finally looping (*lo to ADRESSE times c*). After this loop the pointer has to be set to the next entry in the variable counter list (*ivc*).

Special care for all topspin versions has to be taken for the dummy scans (*ds*), e.g. the instances that the pulse program is executed without recording data. To execute the dummy scans correctly the pulse program requires an acquisition loop with either explicit or implicit (within the *go* command) usage of the parameter *ns* (number of scans). If such a loop is not present in the pulse program, e.g. if the counter *ns* is replaced by another loop variable or constant, data acquisition is turned on with the first execution of the pulse program and thus is on during the dummy scans leading to artefacts in the first recorded FID. Therefore *ds=0* is required in topspin 1x and 2x to acquired the weighted data. In case the dummy scans are required to reach a steady state before data acquisition, they need to be added as explicit loop above the actual pulse sequence. In topspin 3, the explicit loop can be circumvented by introducing an if statement that executes the data acquisition loop for the first indirect point *ns* times and uses the variable counter as loop counter from the second FID on. In topspin 4 it is possible to change *ns* during the execution of the pulse program. This simplifies the implementation compared to topspin 3: the *go* command of the pulse program remains unchanged, the loop commands only (F1PH, F2EA) require the insertion of *calcl* and *ns=WDNS[COUNTER]* instead of *l7=*... (see below).

As an example we list the topspin 3.5 modifications in the Bruker standard pulse program *hncagp3d* below.

;hncagp3d

;avance-version (15/02/27)

…

…

define list<loopcounter> WDNS=<$VCLIST>

"l6=td2"

"l8=0" ;inner loop fid counter

"l9=0" ;outer loop plane counter

"ns=WDNS[0]"

aqseq 321

…

…

d1 pl1:f1

"COUNTER=l8+l6*l9"

"l7=WDNS[COUNTER]"

…

…

if "COUNTER == 0"

{

goscnp ph31 cpd3:f3

3m do:f3

3m ipp3 ipp4 ipp5 ipp6 ipp8 ipp31

lo to 2 times ns

}

else

{

goscnp ph31 cpd3:f3

3m do:f3

3m ipp3 ipp4 ipp5 ipp6 ipp8 ipp31

lo to 2 times l7

}

d11 do:f3 mc #0 to 2

F1PH(calph(ph4, +90) & exec(rppall) & calcl(l9,1), caldel(d0, +in0))

F2EA(calgrad(EA) & calph(ph6, +180) & exec(rppall) & calclc(l8,1), caldel(d10, +in10) & caldel(d29, +in29) & caldel(d30, -in30))

exit

…

…


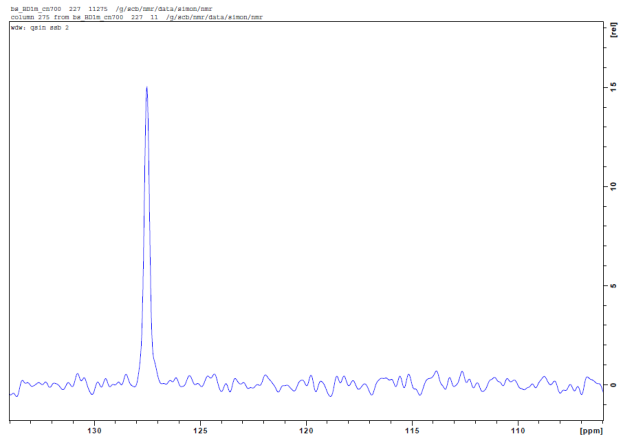

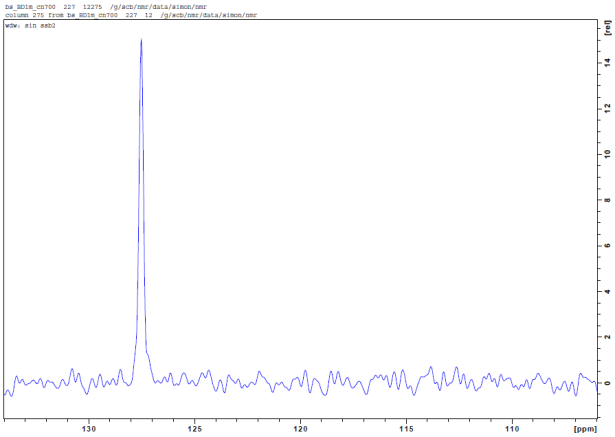

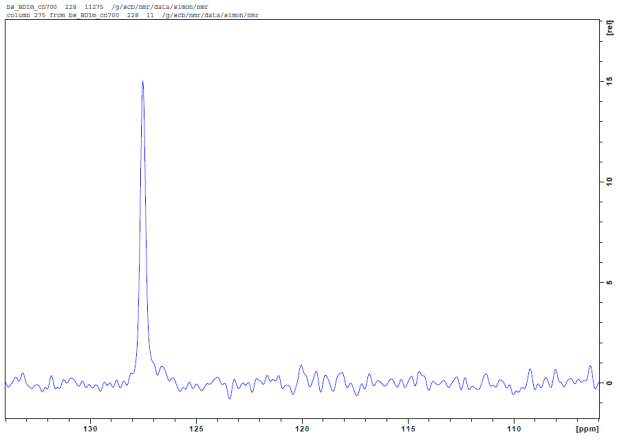

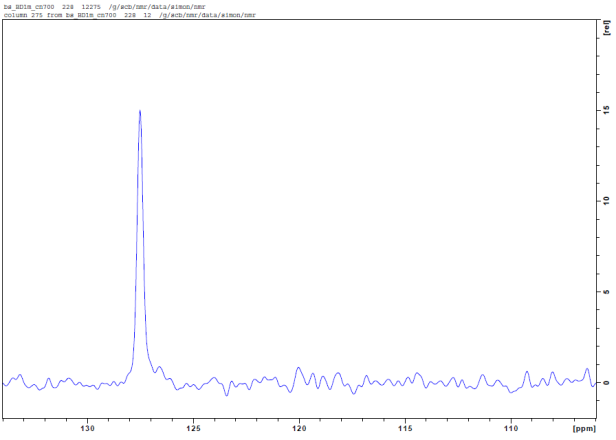


**B**

**D**

**C**

**A**

**Fig. S1** Comparison of one dimensional traces extracted from ^15^N, ^1^H water-gate HSQC spectra used for the comparison in Table 1. **Top**: US reference 1, **A** spectrum processed with cos^2^ apodization, **B** spectrum processed with cos apodization. **Bottom**: cos^2^ WS (first line in table1), **C** spectrum processed with cos^2^ apodization after scaling with w^-1^, **D** spectrum processed with cos^3^ apodization after sacling with w^-1^.

The peak shape of the US data with cos^2^ apodization and the cos^2^ WS are identical (**A** and **C**). The noise floor frequency characteristics of the US data with cos apodization and the cos^2^ WS data are similar (**B** and **C**). If the cos^2^ WS data are additionally apodized with cos, the noise floor frequency characteristics is similar as for US data with cos^2^ apodization (**A** and **D**). The line-width of the signal increases with the exponent α of the cos^α^ apodization (B α=1, A C α =2, D α =3).


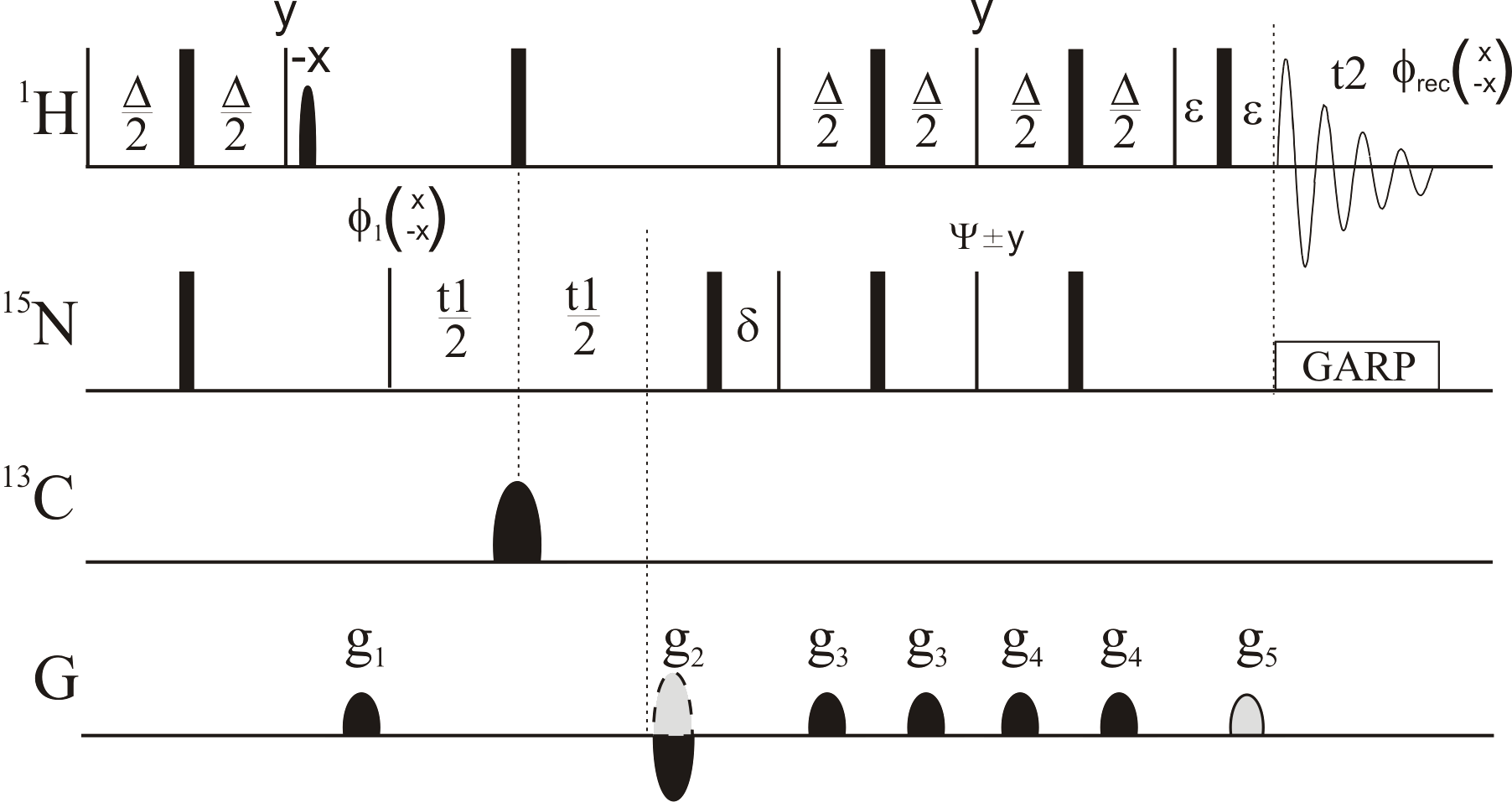


**Fig. S2** Sensitivity enhanced Echo-Antiecho HSQC pulse program. Narrow/wide rectangular bars denote 90˚/180˚ hard pulses. The 13C pulse is a 500μs adiabatic Chrip pulse. Pulsed field gradients were applied with a smoothed square shape (SMSQ) of 1ms duration and g1m=70%, g2m=90%, g3x=5%, g4y=7% and g5m=9.1% of the maximum strength on a triple axis gradient coil along the direction indicated by the letters m(magic angle), x(x-axis), y(y-axis). g2 is inverted for each alternating scan.


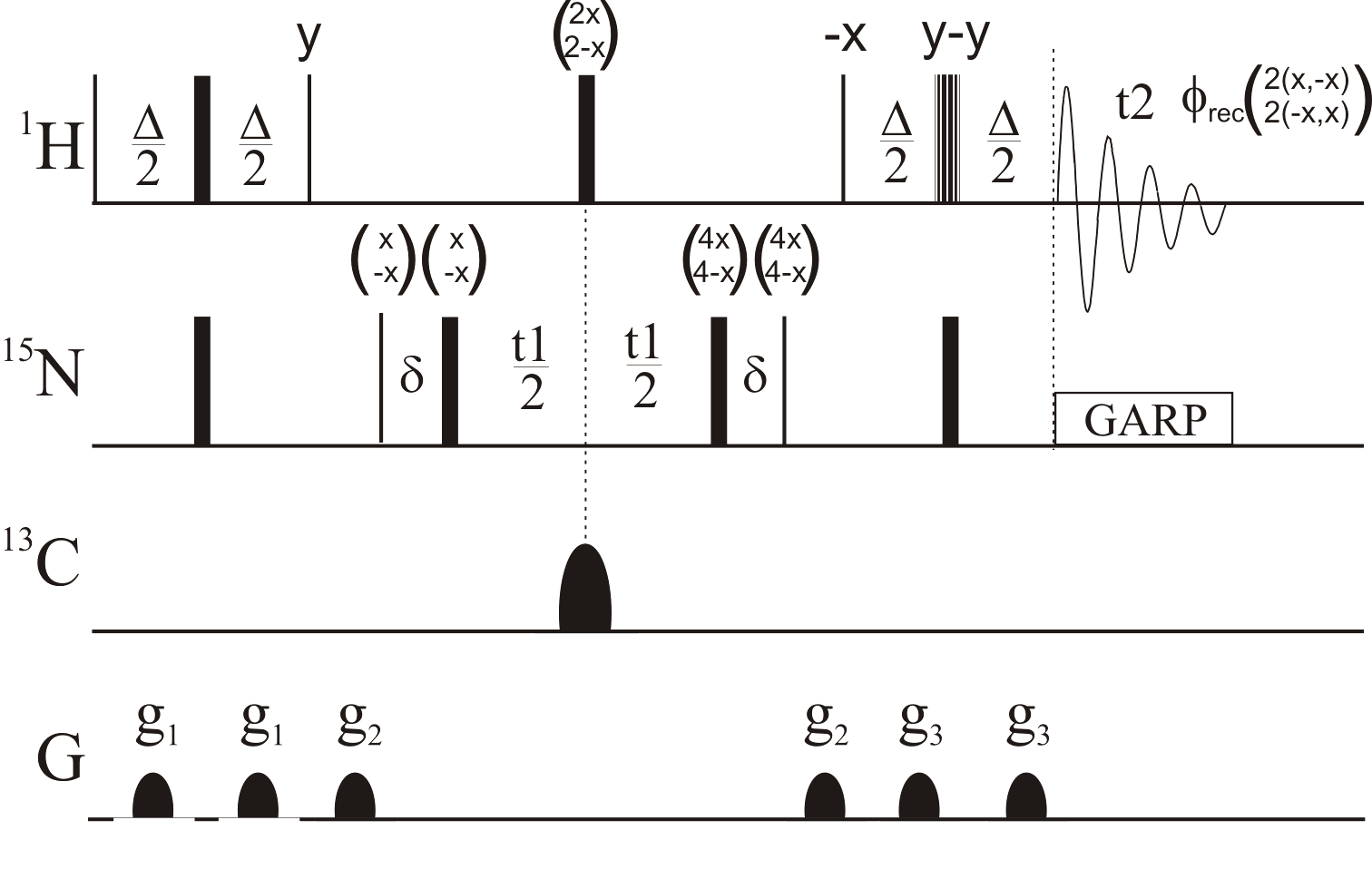


**Fig. S3** HSQC water-gate pulse sequence (fhsqcf3gpph). Narrow/wide rectangular bars denote 90˚/180˚ hard pulses. The last proton pulse is the 3-9-19 water-gate pulse with a 128.8μs delay. The 13C pulse is a 500μs adiabatic Chrip pulse. Pulsed field gradients were applied with a smoothed square shape (SMSQ) of 1ms duration and g1=16.2%, g2=80% and g3=50% of the maximum strength along the z-axis.

**
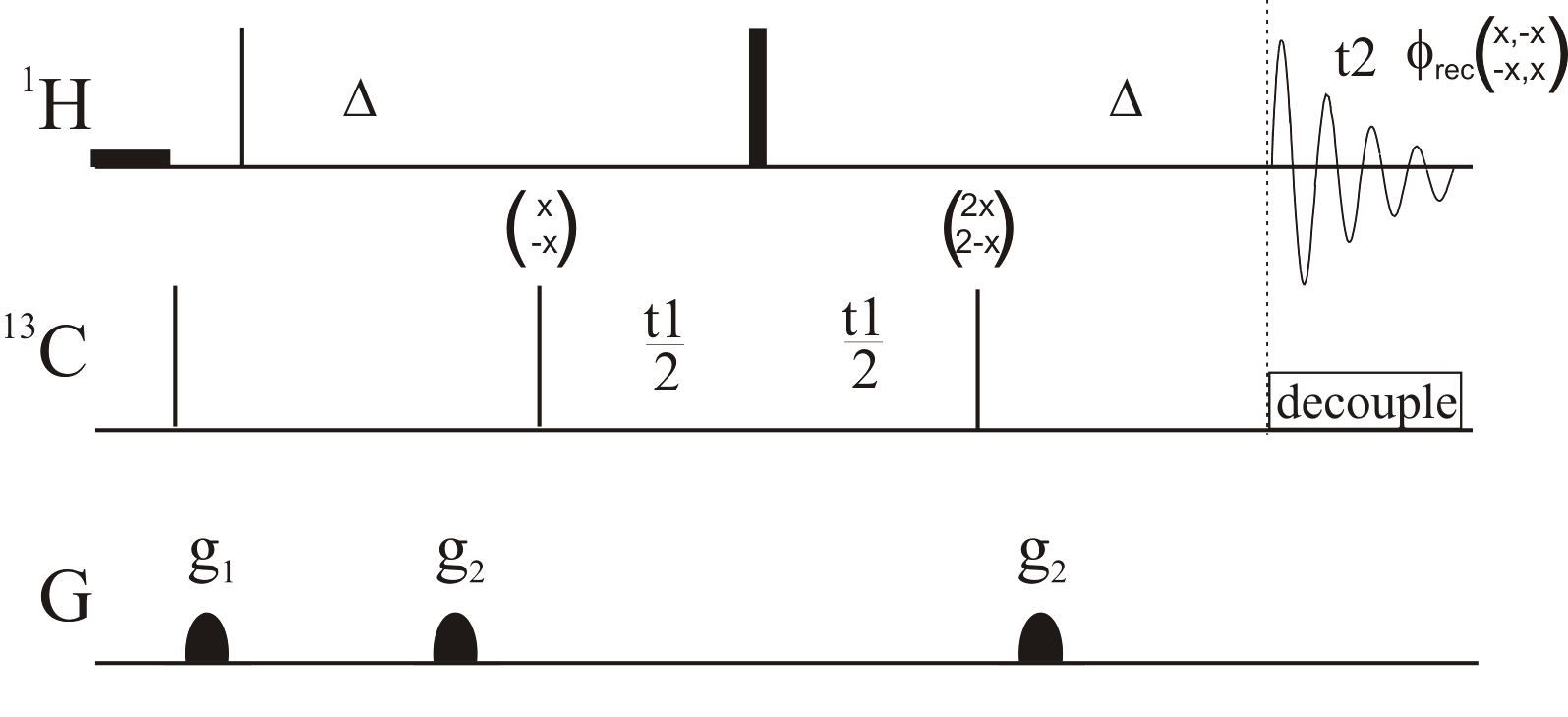
**

**Fig. S4** HMQC pulse program. Narrow/wide rectangular bars denote 90˚/180˚ hard pulses. The small filled rectangle on 1H denotes presaturation of the water resonance during the recycling delay. Pulsed field gradients were applied with a smoothed square shape (SMSQ) of 1ms duration and g1=70% and g2m=90%of the maximum strength along the z-axis.
